# Experiences of seeking and accessing medical care among persons with major depression: A qualitative descriptive study of persons with depression in China

**DOI:** 10.3389/fpsyt.2023.1092711

**Published:** 2023-02-10

**Authors:** Yan Hua Zhou, Doris Leung, Jian Kui Lin, Li Chan Hu, Xiao Yang Lin, Xuelin Zhang, Yim Wah Mak

**Affiliations:** ^1^School of Nursing, Guangzhou Health Science College, Guangzhou, China; ^2^School of Nursing, The Hong Kong Polytechnic University, Hong Kong, Hong Kong SAR, China; ^3^The Affiliated Brain Hospital of Guangzhou Medical University, Guangzhou, China

**Keywords:** depression, seeking medical help, medical care, experience, qualitative study, treatment delay, China

## Abstract

**Introduction:**

A large number of people in China are affected by depression, yet tend to delay seeking treatment. This study aims to explore persons living with depression and their journey of diagnoses and seeking professional medical help in China.

**Methods:**

Semi-structured interviews were conducted with 20 persons who visiting physicians to be diagnosed and receive professional help from a large mental health center in Guangzhou, Guangdong province, China. Individual interviews were conducted and data were analyzed using content analysis.

**Results:**

Three themes were identified from the findings: (1) “noticed something was wrong”; (2) negotiated decisions with their own narratives and the personal suggestions of others; and (3) gave new meaning to their experiences of depression, whereby they sought medical treatment.

**Discussion:**

The findings of the study indicated that the impact of progressive depressive symptoms on the participants’ daily lives was a strong motivation for them to seek professional help. The obligation to care for and support their family prevented them from initially disclosing their depressive symptoms to family members, but eventually prompted them to seek professional help and persist in follow-up treatment. Some participants experienced unexpected benefits (e.g., relief at no longer feeling “alone”) during their first visit to the hospital for depression or when they were diagnosed with depression. The results suggest a need to continue to actively screen for depression and provide more public education to prevent negative assumptions and reduce public and personal stigmatization of those with mental health problems.

## 1. Introduction

Major depression, also known as depression, clinical depression, or major depressive disorder, is a medical diagnosis for a mental illness that should not be confused with normal emotions such as sadness and grief (1). Sadness or grief is a normal response to loss, disappointment, or other difficult experience. Feeling sad or “down” at times, “down” is a normal life experience. People experience differed emotional response patterns of depression, sadness, and grief in life events (2). Usually, when the difficulties are resolved and the person can go about their lives, the feeling of sadness goes away. However, if the sadness does not recede, the person may be at risk for depression (3). Thus, sadness is an integral part of depression (4), though the diagnosis of depression also requires the presence of a loss of interest or pleasure, feelings of guilt or low self-worth, disturbed sleep or appetite, feelings of tiredness, and poor concentration, for at least two consecutive weeks (5). Globally, 4.4% of the population was diagnosed with depression in 2015–an increase of 18.4% compared to 2005 (6). In China, the number of people affected by depression has been increasing year by year. Specifically, the number of deaths or injuries caused by depression rose to a 29-year high in 2019, at more than 50 million (7).

Depression has negative effects on both persons diagnosed and society. In addition to physical discomfort such as chronic pain (8) and sleep disorders (9), persons’ work performance (10) and other social activities are greatly impaired by depression (11). Moreover, depression is one significant factor influencing people to attempt suicide. The WHO (World Health Organization) released data in 2021 indicating that over 700,000 persons with depression commit suicide each year (12).

Depression is also associated with increased utilization of healthcare services, loss of work productivity, and economic costs (13, 14). From 1990 to 2017, depressive disorders, mostly comprised of major depression, rapidly climbed to third place in the list of causes of the number of years lived with disability (YLDs) around the world (15). Effective treatment for depression could be helpful to improving patient outcomes and reducing economic, social, and healthcare costs (14).

Unfortunately, delays in seeking treatment are common among persons with major depression. A national study in the United States (US) reported that the mean time from the onset of depression to the first treatment contact was 47.5 months (16). In Tokyo, the median duration of time during which depression went untreated was 4 months (17). Early treatment has been shown to have the advantages of shortening the length of a depressive episode in recurrent depression (18) and promoting remission (19). Further delayed treatment prolongs the duration of untreated depression, significantly weakening the effect of using antidepressant medications (20).

Several studies have previously been conducted to examine factors contributing to delays in seeking treatment by people with depression. For example, a multinational survey conducted in six western countries, namely, Spain, Israel, Australia, Brazil, Russia, and the United States, reported that barriers preventing persons from seeking treatment for depression included the affordability of and access to psychiatric services, disease-related stigma, lack of social support, and concerns about the side effects of antidepressants (21). Another survey conducted in Australia (22) revealed that self-stigma and perceived discrimination from professionals and others were important deterrents to seeking care among persons with depression. A qualitative study of Latinos in the United States revealed that factors that facilitate or discourage persons from seeking medical care include concerns about privacy and discrimination, language and culture difficulties, financial pressure, factors involving contact with healthcare providers, stigma and isolation, knowledge about depression, and family support (23).

In all previous studies, stigma associated with mental illness appears to dominate as an influencing factor for those delaying seeking medical treatment. Unlike sufferers of other psychiatric problems, such as mania, persons with depression have been reported to conceal their symptoms so that they can make themselves look “normal” (24). This creates a barrier to seeking the medical help crucial to diagnosing and accessing therapy for depression. However, there is limited understanding of the experiences of persons and how they actually seek and access medical help. Improving this understanding could be valuable in providing support for their treatment, and in helping them to access professional help.

There may be different barriers in developing countries to seeking and accessing treatment for depression. In South Africa, younger people (ranging from 18 to 40 years of age), those with other chronic illnesses like tuberculosis, or those with more social support are more likely to seek help for their depression (25), while low confidence in one’s ability to cope with the condition by oneself or with the help of other non-professionals, a low level of literacy in depression, the inaccessibility of medical services, and disease-related stigma were the reasons reported by those with depression to explain why they did not visit doctors (25).

Similarly in China, delays in treating major depression have mainly been due to limited awareness of the problem, limited health personnel to treat the problem, and stigma surrounding mental illness. However, progress has been made in increasing the number of psychiatric facilities (by 77.94% from 2010 to 2015) and the number of psychiatrists and psychiatric nurses (by 47.08 and 110.40%, respectively, from the end of 2011 to 2015) (26). Furthermore, the National Health Commission in China set up a scheme in 2018 to establish a country-wide psychosocial support system (27). The scheme provides mental healthcare to a greater number of people in rural areas, pre-school children, and university students on campus. Yet, from 2002 to 2003, only about 6% of persons in metropolitan China sought treatment for mood disorders, including major depressive disorder, in the same year as the onset of their disorder (28). A survey conducted from 2001 to 2002 in the two biggest cities in China, Beijing and Shanghai, revealed that only 22.7% of persons with depression sought medical help within a year (29), as compared to 51.9% in the US (16) and 70% in Japan (17). Why people in China continue to delay seeking help for depression is a question that remains to be explored.

People in developing countries like China, may exhibit specific belief patterns related to seeking and accessing treatment for depression. Jiang and colleagues conducted a survey of 20 primary care clinics in Hangzhou, the provincial capital of a developed province in China. They found that only 7.5% of participants believed that depression is a disorder that should be treated by healthcare professionals, while 32% thought that depression is the individual’s fault (30). The significant percentage of people in China interpret depression as individual’s fault may related to the lower level of depression literacy among Chinese population than other culture groups (31). On the other hand, traditional Chinese philosophy suggests that people with mental illness is punished for the wrongdoings of their family members or ancestors (32). Furthermore, Chinese people with depression may deny their condition or not recognize it in expressions of their bodily symptoms (33). A survey by Wong et al. conducted in Shanghai reported that only 12.3% of participants could properly identify symptoms of major depression that were presented in a vignette, and fewer Shanghai Chinese than Chinese living in Hong Kong or Australia believed that social and family support are helpful for persons with depression (31). In other words, ethnic and contextual differences might influence the way persons interpret depression and their perspectives about its treatment, or even their attitudes toward seeking mental healthcare (34).

Few studies have been carried out to explore how people in China understand and recognize mental illness, and what their preferred treatment might be. In view of this, the present study aims to shed light on the experiences of persons in China who had been diagnosed and were living with major depression, and how they seek and access professional medical help.

## 2. Materials and methods

### 2.1. Purpose

To understand how persons in Guangzhou, China who have been diagnosed with major depression:

(1) Experience living with major depression.

(2) Seek (and make decisions about) professional medical ser-vices, and are assisted or hindered in accessing such services.

### 2.2. Study design

The authors adhered to a study design of qualitative description, which it has been suggested is appropriate for exploring experiences and insights from informants regarding an understudied phenomenon (35). Further, a post-positivist paradigm was used to obtain “a comprehensive summary of events in the everyday terms of those events” (P334) from participants (36). In other words, data were collected directly from participants with a rich knowledge of the phenomenon, who were assumed to be socially constructing their beliefs and values about the phenomenon, in relation to the context in which they were interacting with the researchers (37).

### 2.3. Sampling

Participants were recruited from the Outpatient Department in The Affiliated Brain Hospital of Guangzhou Medical University, the largest such institution in Southern China, which provides mental healthcare services to people from the surrounding regions of Guangdong, Guangxi, and Hainan. Specifically, the Center provides medical consultations and care for adult persons with mental disorders such as bipolar disorder, major depression, obsessive-compulsive disorder, social phobia, and anxiety disorders. Among all persons who use mental healthcare services in the department, 20% have been diagnosed with depression.

Purposive sampling of participants who were willing to participate included those who fulfilled the following criteria: (1) received a primary diagnosis of major depression from a physician; (2) in a stable condition (as assessed by a physician) for at least 1 month post-discharge, if previously hospitalized; (3) self-identifies as ethnic Chinese; (4) speaks fluent Mandarin or Cantonese; and (5) ≥18 years old.

Participants with the following characteristics were excluded: (1) unable to articulate their thoughts; (2) diagnosed with major depression as part of a bipolar disorder; and (3) diagnosed with depression secondary to other primary psychiatric or physical conditions.

### 2.4. Data collection

Data were collected with the help of the medical team of The Affiliated Brain Hospital of Guangzhou Medical University. Data collection took place over a 1-year period from December 2020 to December 2021 during the Coronavirus pandemic (COVID-19), when the Center had visiting restrictions. When persons came to the Center for a medical consultation, two psychiatrists screened potential participants for eligibility. The researcher invited eligible and interested persons for an interview, and explained the objectives and procedures of the study, after which their informed consent was obtained. Guest et al. (38) suggested that a sample size of 12 is adequate to understand common experiences from a relatively homogeneous population. Hence, it was anticipated that inviting a total of 20 individuals to participate in the study would be enough to meet the requirements for saturation, as the selected population was relatively heterogeneous.

Face-to-face interviews were carried out using a semi-structured interview guide. These included open-ended questions such as: “Would you tell me about how you came to be diagnosed with depression?,” “Could you tell me about your experiences of seeking professional help for your depression?,” “What were the difficulties that you encountered when seeking or accessing professional healthcare services?” Prompts such as “Can you talk more about.?,” “What was it like for you when…?,” “What do you mean when you say…?” were raised as appropriate. At the end of the interview, the participants were given a summary of their narrative, and invited to confirm or clarify it and add anything to their interview.

The interviews were conducted by the first author (YZ), a female nurse educator with experience in interviewing persons with depression. The interviews took place in the nurses’ office in the outpatient department or elsewhere in the hospital as suggested by the persons. Both parties wore masks during the interviews, according to COVID-19 safety guidelines. All of the interviews were conducted in Mandarin and were recorded using a voice recorder pen and transcribed verbatim. The interviews took an average of 30–45 min to conduct.

### 2.5. Data analysis

The method of conventional content analysis (39) was adopted when analyzing the data to get at its manifest content, “close to the text”; as well as latent content, reflecting the participants’ underlying meanings of their experiences (40). First, the recorded interviews (the unit of analysis) were transcribed verbatim by two researchers (LH and XL), both of whom had more than 5 years of experience in working with persons with depression, and had been trained to consistently transcribe the records. One of the researchers (YZ) checked the transcripts against the original audio recordings for accuracy and read the transcripts back to become familiar with the data. Second, two researchers conducted an inductive content analysis, starting with open coding. Upon reading each transcript, the researchers wrote notes and “named” meanings reflecting a “code” in the text, which were collected in “coding sheets.” Then, they grouped the codes into patterns, which generated sub-categories. Finally, these sub-categories were further grouped into prominent ideas, reflecting “main categories.”

In order to establish rigor in the data analysis, criteria on credibility, plausibility, and transferability were chosen, and strategies to meet each were employed (41). Throughout the study, contextual field notes, methodological notes, analytic notes, and notes on self-awareness (reflexivity) were recorded. To begin with, one researcher (YZ) carried out an independent analysis of 20 interviews to establish credibility and confirmability. A second researcher (YM) then checked the initial coding (identified as “meaning units”) and determined whether there were any conflicts of interpretation. Credibility was further established through a prolonged effort to comprehend and confirmed the interview data by two researchers (YZ and YM). All codes were discussed to reach a consensus on the patterns that had been grouped into categories and subcategories. Data saturation was confirmed by three investigators (YZ, YM, and DL), when they agreed that no new codes had emerged after 20 persons had been interviewed. To ensure that the codes were credible and plausible, all analyses were performed using Chinese transcripts, after which relevant quotes were translated into English for reporting purposes. Two bilingual Chinese–English translators validated the translation of those quotes (YM and XZ). A third researcher (DL) joined the team to re-contextualize sub-categories and categories for plausibility. Two researchers (YM and DL) then further checked the data against the preliminarily identified categories to further enhance the credibility of the data. Finally, exemplar quotations were identified for transferability by readers, in the final dissemination of this report.

### 2.6. Ethical considerations

Ethical approvals were obtained from the Ethics Committee of The Affiliated Brain Hospital of Guangzhou Medical University and The Hong Kong Polytechnic University. Three authors of this study facilitated the recruitment of participants (JL, LH, and XL), as they were nurses in the hospital (one female nurse manager and two female staff nurses), although in order to avoid a conflict of interest, none worked directly with the persons. Further, the persons were assured that their participation in the study was voluntary, and that they could withdraw from the study at any time without any negative consequences to their treatment in the hospital. Participant information containing any personal identification was stored in an encrypted USB, password-protected for online transfer, and was accessible only by the researchers in this study. During the interviews, the researchers were alert to signs of distress that might cause any participant to be unable to continue with the interviews, but none were observed to have occurred.

## 3. Findings

### 3.1. Characteristics of the participants

Twenty people participated in the study, ranging in age from 18 to 59 (*M* = 25.75, *SD* = 9.32). Three were males, while 17 identified as female. The length of time that the persons had spent living with depression ranged from half a year to 20 years. To understand their delay in seeking treatment, each participant was asked, “How long was it between the onset of your depressive symptoms and your first visit to a doctor to address the symptoms?” We found that delays in seeking mental healthcare were prevalent, as only one participant had visited a doctor within a week of the onset of his/her depressive symptoms. To calculate the interval between the onset of depression and the persons’ first visit to a doctor for mental healthcare, we rounded the length of time up or down to the nearest half year. Characteristics of the participants’ history of depression are summarized in Table 1.

**TABLE 1:** Characteristics of the participants’ history of depression (*N* = 20).

Characteristics	Range	Mean	SD
Age of onset (year)	12∼53	17.90	8.6
Length of time living with depression (year)	0.5∼20	7.85	5.16
Interval between the onset of depression and the first visit to a doctor for mental healthcare (year)	0∼11.5	3.43	3.17

### 3.2. Categories

**FIGURE 1:**
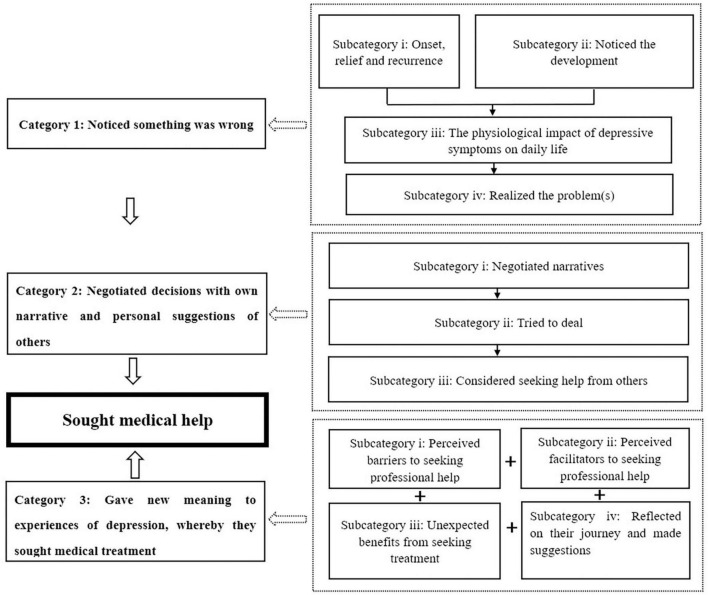
The road map of the persons’ decision-making process.

The participants recounted their experiences from first becoming aware of a “problem” in their daily activities, trying to solve the problem, seeking medical help, and being diagnosed with depression. Three major categories reflected their decision-making process (see Figure 1). These were the ways in which the participants: (1) “noticed something was wrong,” characterized by four subcategories; (2) negotiated decisions with their own narrative and the personal suggestions of others, characterized by four subcategories; and (3) gave new meaning to their experience of depression, whereby they sought medical treatment, characterized by four subcategories.

#### 3.2.1. Category 1: “Noticed something was wrong” (having problems)

Participants in the study realized that “something was wrong” when they first noticed “problems/mistakes” and how these “problems/mistakes” affected their lives before seeking medical help. Four sub-categories emerged during a further analysis of this category: (i) onset, relief, and recurrence of depressive symptoms; (ii) depressive symptoms: behavioral changes, increased negative emotions, and hallucinations; (iii) the physiological impact of depressive symptoms on daily life; and (iv) the realization that the problem(s) is/are serious and that one must seek medical treatment.

##### 3.2.1.1. Subcategory i: Onset, relief, and recurrence of depressive symptoms

Throughout the journey of depression, most participants experienced a pattern of onset-relief-recurrence. The initial onset of depression: Participants described their first awareness of depression as having occurred under various stressors, such as schoolwork, peer aggression, conflict, or a family problem.

P6 recalled that she was under a lot of pressure over her final exams: *“Because it happened around the end-of-term exams, at that time I thought that it was just because I didn’t know how to relieve the tension. So I didn’t doubt that I had some mental issues.”*

P8, who was [verbally] attacked by classmates when she was in elementary school, reported: *“Because at that time I was*… *not only at home*… *depressed and miserable, but also at school*…. *I very sad, I was ostracized by the whole class at school*… *for, um*… *that is*… *this is what happened when I was in the sixth grade.”*

One female (P10, 59 years old) believed that her depression was triggered by her son’s divorce: *“(In 2015), I felt that there were already some signs, because at that time my son. divorced, and after he divorced, I was a little worried about my grandson (about who would take care of him), and it was easy to cry.”*

The symptoms abated, but then intensified with new stressors, causing a relapse: Some participants said that their symptoms of depression eased or disappeared as the stress was resolved; however, they reappeared and worsened with new life events.

P4 described the disappearance of her depressive symptoms after the college entrance examination, but said that they worsened when she felt various pressures at college. *“Because I felt relived after the college entrance examination, my mood was*… *relatively relaxed, until I entered university, maybe more than half a year ago, and then these problems started again*… *and gradually got worse.”*

P10 stated that her depressive symptoms reappeared because some unexpected changes occurred in her family after she returned from traveling abroad. She said: *“It would come right after traveling. It did seem that I felt a bit relaxed after traveling*… *until October 2016. Then*… *my father had lung cancer, which was in the advanced stage and had spread. At that time, it was very stressful. Taking care of him and looking after my grandson made me feel uncomfortable.”*

A young female participant (P17) experienced a long remission from her depression after graduating from junior high school, but her condition deteriorated rapidly in college when she experienced heartbreak in a relationship: *“I got better when I was in high school*… *but the feeling of being hurt (by my ex-boyfriend) pushed me over the top, just caused me more hurt, so that I beat up on myself more frequently, and even attempted to commit suicide.”*

In our study, younger persons reported feeling a greater obligation to fulfill expectations and not impose an additional burden on their parents. For example, a college student complained about the costly depression treatment, stating: *“[Although you] get financial support from parents, and you prolong your life by taking very, very expensive drugs, you still want to die. Now that you’re free, you would feel [guilt at spending your parents’ money for treatment].”*

##### 3.2.1.2. Subcategory ii: Noticing the development of depressive symptoms, including behavioral changes, negative emotions, and hallucinations

Many of the participants noticed that there were many changes in their behaviors and emotions. For instance, one participant (P4), who was a college medical student, described her experience this way: *“You (I) just can’t see any direction, you (I) can’t see anything*…. *[I was] in a rather desperate situation, then, I was very unhappy every day, couldn’t sleep, and didn’t want to eat or move around.”*

Another participant (P6) said, *“I don’t like to tidy up anymore, as I said before, my desk and everything used to be neatly arranged*… *and same with my home. I was not like this before (I lost interest in clearing up), gradually, it is becoming messy.”*

Several participants had thoughts of self-harm or suicide, and some acted on their intentions.

One 24-year-old participant (P18) who had experienced depressive symptoms for 7 years prior to treatment, reported that: *“Many times [I have] obvious thoughts of suicide, sometimes [I] even think about*… *when and how to end my life.”*

A female participant P9 admitted that: *“Hurting myself (showing the scar on her wrist to the interviewer)*…, *at the beginning, I was*… *taking the eyebrow razor*… *slashing like this (showing the cut on her wrist)*…*.”*

Some participants experienced hallucinations. For example, one participant, P6, questioned her reality with family members when she stated: *“I heard people say those words to me, all abusive words or something similar, and then I really saw a figure of the person behind me.”*

##### 3.2.1.3. Subcategory iii: The physiological impact of depressive symptoms on daily life

All of the participants stated that the effects brought on by depressive symptoms were just not psychologically and emotionally problematic, but physiologically as well. Participants reported symptoms that altered the circadian rhythms of their bodies (i.e., sleep/wake, hormonal cycles), further affecting their performance at school or work, and sometimes even their ability to continue studying or working. For example, a female college student (P4) said: *“It’s not just your (my) mental state, it’s my physical condition, I. um*… *for example. my menstrual period was affected, then I was unable to eat, and I was unable to fall asleep. I just wanted to lie down every day.”*

In a few instances, work was significantly affected by the disease, such as when P16 stated: *“My concentration and memory have declined, especially when I am sometimes attacked*…. *I really don’t want to work, and I often feel very tired.”*

One participant, P8, even quit school in her first year of college due to the severity of her depressive symptoms: *“I told my parents, I said that my current status may not be good, I have to drop out of school.”*

##### 3.2.1.4. Subcategory iv: Realization that the problem(s) is/are serious and that one must seek medical treatment

After experiencing a period of emotional distress and its impact on their daily lives, some participants sought further insight about their illness in their own way. Alternatively, they gained answers indirectly through others such as online sources, or family or friends. All suggested that they were suffering from a mental health problem.

The realization that they had a mental health problem prompted some to wonder whether it would be depression. For instance, P7 said: *“Then, I did search online, I wondered if it was depression, and I checked (my problems) against the symptoms of depression. They seemed to tally with [my experience]. I began to believe [that I was having depression].”*

Sometimes, participants were prompted to seek medical treatment as a result of their parents’ realization. For example, P20 visited a doctor less than 1 month after the onset of her depression. She said: *“My mom raised the possibility that the symptoms point to mental issues.”*

Occasionally, a physician treating their physiological problems might refer them to a mental health professional, such as a psychologist. P13 said: *“I visited the neurology department in 301 (No. 301 hospital). After reviewing the results of my various medical tests, the doctor asked me if I would like to visit the psychology department.”*

#### 3.2.2. Category 2: Negotiated decisions with one’s own narrative and the personal suggestions of others

Throughout the journey of seeking medical treatments, the participants had their own opinions of their “problem(s)” and yet sought out some advice and help from others to help them make sense of their narratives. The participants sometimes accepted help from others, although usually they negotiated the narratives in terms of how they made sense with their own ideas at different stages of seeking treatment. In this process, three subcategories emerged: (i) negotiating narratives when initially aware that something is wrong; (ii) trying to deal with the “problems” in order to avoid making sense of them, and (iii) considering seeking from others to help re-negotiate their own ideas of depression.

##### 3.2.2.1. Subcategory (i) negotiating narratives when initially aware that something is wrong

Although the participants, as described earlier, became aware of their depressive problems and how it affected their daily activities, some could not believe that it was happening to them. For example, P6 said: *“I am doubting my judgment*,… *I used to be really cheerful, sunny.*… *I am just thinking whether people like me really would have this disease? A little weird, is this my problem?”* Some preferred to solve the “problem” by themselves, such as when P7 said: *“I have one set of self-regulation strategies*…. *I could go back to doing what I should do, but I gradually found that these strategies did not work well.”*

The participants considered discussing their problem with family members, but feared that they would not be understood. For example, her parents’ negative attitudes toward the suicidal behavior of others affected P1’s decision. She said: *“Then I thought that my family*,… *who do not understand the disease, might think that I was hypercritical or overthinking*…. *Was not there always a lot of news about suicide before?. Then, they (my family) would say that they (the suicides) can’t figure it out or something like that, when they touch on the topic of suicide.”*

Some participants reported that they had thought of seeking medical help, but hesitated due to a fear of stigma. For example, P9 reported feeling ashamed of visiting a hospital for depression. She said: *“I didn’t ever think of seeing a doctor. I was upset, because*… *as an ordinary person, going to the hospital to have a medical check (for depression) is just uncomfortable*…. *I just felt that things cannot be that bad.”* P17 erroneously assumed that treating depression would be financially costly. She said: *“When I heard [that I had] the disease (depression), I thought that it must be a costly disease. [I should] visit the doctor many times, and it could even cost tens of thousands of yuan. My family’s [financial] situation at that time might not allow me to do that (seek medical help), so I didn’t tell them (parents) and didn’t dare to see a doctor.”*

##### 3.2.2.2. Subcategory (ii) trying to deal with the “problems” in order to avoid making sense of them

In response to the question of how they identified and dealt with “problems,” some participants said directly that they actually did not deal with the depressive problems but just avoided them, to delay making sense of them. For example, P15 tried to distract herself from addressing her depressed mood. She stated: *“It was escaping rather than handling, by (watching) animations, reading, and doing something like that*… *fully devoting myself to other people’s stories.”*

In another instance, P16 chose to ignore the problem. She reported: *“I found that I was ignoring or suppressing them (depressive symptoms) most of the time*…. *[I] did not really want to face it (depression).”*

Similarly, a male participant, P3, said: *“Then, I forced myself*… *to be happy every day, or I to greet people I had met, or something like that.”*

Some participants sought help from doctors in various departments for their physical problems, but avoided making sense of their symptoms of depression. For example, P14 said: *“I visited doctors in other departments before I came to the psychology department. For example, I went to the neurology department for sleeplessness and to the ear-nose-throat department for tinnitus*… *ears, eyes, my stomach, and sleeplessness, I visited different departments for those (problems).”*

Some participants tried to solve their problem by using Traditional Chinese Medicine. For example, P3 said: “*At that time, I was at home for the first 3 years (because of depression). I used traditional Chinese medicine for a period of time.”*

##### 3.2.2.3. Subcategory (iii) considering seeking help from others to help re-negotiate their own ideas of depression

Many participants continued negotiating a process weighing the benefits vs. the costs of treatment. This helped them to re-negotiate the meaning of receiving a diagnosis of depression. For example, P1, who was a third-year senior high school student, weighed the effects of her symptoms on her studies against the cost of treatment. She reasoned: *“If I didn’t see a doctor, my future would have been affected, so*… *comparing the money (for treatment) with the wages I will earn in the future, the gains in fact outweigh the losses. So I decided to see a doctor after making such a comparison.”*

In comparison, concerns about family helped the participants to re-negotiate the seeking of medical treatment for their depressive symptoms rather than acting on thoughts of suicide. For instance, P3 explained: *“I feel that family is still so important*…. *It was not long after my father died, and my dad’s death made me*… *so unhappy*… *so depressed. If I were also to die, my mom and my grandma might lose the will to live, so I feel that I must live no matter what.”*

In another example, P7’s treatment-seeking behavior was reportedly encouraged by her friends: *“Anyway, what they said was that early detection and early treatment were important, and (I should) see a doctor soon. Maybe I would have delayed [consulting a doctor] if I had been alone, but they encouraged me like this and I thought that it would be better to see a doctor earlier.”*

#### 3.2.3. Category 3: Gave new meaning to experiences of depression

Four subcategories characterized this main category when participants demonstrated a process of giving new meaning to their experiences of depression through: (i) perceived barriers to seeking professional help; (ii) perceived facilitators in seeking professional help; (iii) unexpected benefits from seeking treatment; and (iv) reflecting on their journey and suggestions for others with depression.

##### 3.2.3.1. Subcategory (i) perceived barriers to seeking professional help

On the journey of seeking medical help for depression, there were various obstacles that prevented the participants from seeking early medical help and continuing their treatment. Participants encountered multiple obstacles, from themselves and others close to them. For instance, some reported lacking literacy about depression. One example was P17, a junior high school student who first reported symptoms of depression as something foreign to her. She said: *“I had never heard [of] depression*…. *[I] didn’t know about depression; [I] didn’t know I was sick.”* Some participants felt that the physicians were medicalizing their situation. For instance, P8 had a positive attitude toward medical treatment and the future at the time of her interview, but she admitted that: *“Unable to avoid being depressed, down, and anxious, at that time I still wanted to quit.”*

For some participants, expressions from family members about the stigma of depression was a strong deterrent. For example, when P9 expressed a desire to go to a psychiatric hospital for her depressive symptoms, her parents opposed her decision. P9 stated: *“I originally wanted to go to the Nanchang psychiatric hospital, but they (my parents) said that it did not seem to be such a big deal. They believed that visiting a psychiatric hospital [was a loss of face].”*

Furthermore, practical barriers delayed their seeking and/or continuing to access medical help, such as: a lack of time, too far a distance to travel, and financial concerns. P13 complained: *“Since it’s really too far away (from my hometown), and really too inconvenient*… *[and I face] financial pressures, I can’t come for a follow-up often, which I think delays my recovery.”* In particular, younger participants, like P1, who was a senior high school student, did not wish to burden her family with the costs. She stated: *“I can’t afford it by myself*… *or I don’t want my family to spend a lot of money, so I am thinking of saving money by myself and seeing a doctor in the coming winter break.”* In these instances, some persons expressed no motivation to take medical treatment during their depressive episodes. For example, P18 stated: *“I knew I needed to get moving, but I really had no motivation to take action.”*

Altogether, the majority of participants reduced or stopped taking medications by themselves due to the actual or anticipated negative side effects. For instance, P10 did so after experiencing side effects from the treatment and fearing its long-term consequences: *“I was just afraid that taking too much medication would make me dull because, after all, these are medications. As long as it is a medicine, it has to have some components of poison (a Chinese saying).”*

Similarly, P14 expressed the worry that the medications would be detrimental to her work in dealing with customers in her family’s (online) business: *“But when I am going to work, I absolutely refuse to take the medication because it makes me sleepy.”*

In addition to the side effects of the medications, some participants stopped taking their medication when their depressive symptoms were resolved and they thought that they no longer needed it. For example, P20 stated: *“Obvious sleepiness, lack of energy, feeling down, poor concentration – I felt that the symptoms had cleared up, so I stopped taking medications.”*

When participants needed to take medications for their physical problems, they would put a stop to their medications for depression to prevent the drugs from interacting. For instance, P8 reported*: “If I had a cold, I would take medications for my cold for a few days (and stop my medications for depression). I would continue to take the medications (for depression) after the cold is gone.”*

P3 reduced his dosage of medication fearing that he would not get medications for depression since the hospital had visiting restrictions during the COVID-19 epidemic. He said: *“The epidemic was not over. I didn’t know when I would be able to get the medicine from the hospital*… *(so) I reduced the dosage of the medicine by myself.”*

Moreover, some participants expressed a sense of mistrust of their patient-physician relationship, such as in relation to their medical diagnosis and treatment. For example, P7 expressed mistrust of how his physician arrived at the diagnosis of depression when he stated: *“I filled in several questionnaires during my first visit to the hospital, and I doubted whether depression could be diagnosed by filling in the questionnaires.”*

In other examples, doctors were perceived as not being empathetic to the persons’ situation, and not addressing their specific needs. For example, P4 reported: *“Maybe I haven’t established a good relationship with the doctor*… *a good relationship of trust*…. *Maybe in my view he didn’t address my needs*…. *S/he just asked how I was doing, uh*… *can I sleep, can I eat*… *routine questions*…. *S/he didn’t listen to me carefully*…. *I just felt that I went to the follow-up only to get medications” (P4).*

Similarly, P9 thought that the doctor didn’t take individual differences between persons into account when prescribing medicines. P9 stated: *“Then, s/he prescribed the same medicine for us (a fellow student with a different condition and me). It seemed that s/he was doing it wholesale*…. *S/he prescribed it once every four months. I will only see the doctor three times a year. How could s/he have exact knowledge of my condition (during the following four months)?”*

Moreover, some participants feared that healthcare professionals might judge them harshly. In one instance, P13 shared her unpleasant memories of being a home wrecker and was criticized by her doctor. She reported: *“But s/he would criticize me*… *s/he would say, how can you do this? Why did you do that?”* Other participants anticipated negative judgments if they did not report that they were responding well to the medical treatment. For instance, P5 expressed a negative attitude about his treatment when he thought there was no improvement. He reported: *“There was no difference whether or not I took the medicine. It was just useless.”*

##### 3.2.3.2. Subcategory (ii) perceived facilitators in seeking professional help

Family and peer support were important facilitators in encouraging the participants to seek professional treatment and persist in continuing treatment.

For example, P1 was greatly encouraged by parental support, even though she thought that her parents could not understand her situation and she did not tell them about her condition. She said: *“My parents*… *were the first to support and encourage me, asked me to see a doctor and telling me to take medications with minimal side effects, which I felt was very heartwarming.”*

In another example, P17, who was a female college student, benefited from advice given to her by her friends. She recalled: *“They (friends) persuaded me to see a doctor. I had not known that I had major depression until I visited (the doctor).”*

Family and friends, and the effect of the disease itself on their lives, motivated the participants to seek medical help and follow through with their doctor’s prescribed treatment/plans. For instance, although P18’s condition did not improve after many years of treatment, she continued to persist in the hope that she would make her family happy when 1 day she did recover. She said: *“My family and my mom would be very happy if I am well.”*

Further, participants were motivated to seek professional help to maintain their hope for their future, whether to help themselves or a family member. For example, P4, who was a fourth-year college student, faced pressure to get well in order to graduate and find work. She explained: *“I care about my future, and if I can make money, I do not think I will come to see (a doctor).”* P5’s pet cat motivated him to continue his treatment, as he said: *“I had to make money to vaccinate it (his cat), and I should make money to have it neutered.”*

In one case, a participant talked about wanting to be a good role model for her friend, who also had depressive symptoms. P9 reported: *“My friend once stopped taking medicine*…. *I would like to stand by her [and set an example for her], [let her know] that if I can persist in taking medicine, so can she.”*

A few participants persisted in taking their medication in order to prevent effects if they stopped. For example, P2 said: *“There would be an abstinence reaction if I didn’t take this medicine. I dare not stop (taking the medicine).”*

##### 3.2.3.3. Subcategory (iii) unexpected benefits from seeking treatment

Although most participants delayed their first visit to the hospital to seek professional help, all received unexpected benefits from seeking treatment. For instance, one young male participant (P3) was greatly relieved to no longer feel “alone” in his suffering: *“I think it turns out that I am not alone fighting [depression] in the world. There are lots of people fighting alongside me (laughed).”*

In the process of recognizing others with depression, P17 came to realize the importance of seeking early treatment: *“It turns out that it was not just me who had depression – lots of people had it, but they had all looked for medical help*…. *I think I am too late.”*

Over time, some participants were able to let go of their burden of stigma when they experienced a sense of peer belonging during their visits to the hospital. For example, P13 emphasized the importance of a mental health specialty: *“People like us still have a place to [seek help], which specializes in helping us. There is no need to despair or regard yourself as a marginal person or an outlier.”* The camaraderie with peers allowed some participants, like P20, to stop blaming herself for her diagnosis of depression. She stated: *“[I] found the exit, [it was] not my problem; it is a physical problem indeed, or an issue with my nerves.”*

##### 3.2.3.4. Subcategory (iv) reflecting on their journey and suggestions for others with depression

Many participants emphasized the importance of seeking help from others, especially the importance of early treatment and persisting with treatment to avoid actions that one might regret. For example, P12, who was saved by her schoolmates when she threatened to harm herself, stated: “*No matter how scared you are, you should talk to your classmates, talk to your family, and ask them to help you.”*

When the participants were asked if they wished that something had been different in their journey, P8 stated: *“I especially wish that I had persisted in taking medicine when I was moderately depressed*… *it would not be as severe as it is now.”*

Another participant, P15, commented that she wished that she had sought treatment sooner than 6 years ago. She said: *“If I had been treated earlier, everything would be different now.”*

In summary, a process of giving meaning to the participants’ suffering occurred in a journey of challenges and unexpected benefits, from family and friends, to overcome personal and public stigma and low health literacy about depression and its medical treatment.

## 4. Discussion

The purpose of this study was to explore the persons’ experience of living with depression and the journey of seeking professional medical help in China. Three categories emerged in all of the interviews: (i) “Noticed something was wrong,” (ii) Negotiated decisions with their own narratives and the personal suggestions of others, and (iii) Gave new meaning to their experiences of depression, whereby they sought medical treatment. Overall, similar to previous studies (21–23), the findings in our study indicated that treatment delays were common among persons with depression. Doblyte and colleagues conducted a review of studies (42) to discern delays in seeking medical treatment by individuals with depression. They found that seeking help was a threat to ones’ identity, and engendered either conflict or support from one’s social networks. Therefore, persons with mental health problems sought alternative coping strategies that delayed their treatment-seeking behavior (42). This is consistent with the findings in our study, in that the participants delayed seeking professional help because they encountered many barriers to doing so. Similarly, these results resonate with a previous study from the United States of African-American parents of youth with mental health problems, when seeking professional help for them (43). In the latter study (43), parents experienced several stages in decision making, including identifying the problem, denial of the problem, solving the problem by themselves, considering seeking assistance from outside, getting advice and help from others, conducting a personal analysis, and selecting a solution and seeking an evaluation.

As in our study findings, other studies reported that the perceived severity of depressive symptoms on a person’s inability to perform their usual study and work responsibilities was a frequent trigger for persons to seek medical help (44). Colandrea (45) found that college freshmen with moderate-to-severe depressive symptoms showed a significant preference for counseling services compared to those with minimal-to-mild symptoms of depression. Further, persons who complained that depression had a more severe effect on their lives, such as causing sleep difficulties and energy loss, were found to be more likely to seek medical help (46).

Moreover, one’s ethnic background and social context of living influence how people living in China interpret depression in different ways. In general, Chinese persons are more likely to deny depression or express it somatically than non-Chinese (33). Similar to our study, persons initially sought medical help not for depressive symptoms, but more often for somatic symptoms (47). In part, this may have been to avoid judgment from one’s significant others, as well as because of one’s own internalized shame about having a mental health problem. This was shown in a study by David et al. (48) of participants with psychosis, whose significant others often disapproved of their decision to seek treatment. The authors suggested that this may have been due to the cultural stigma associated with psychosis vs. that associated with depression. Ethnic background also influenced their way of help-seeking for depression. Depressed patients in China may seek help from non-psychiatric professionals, such as general hospital services, lay help, alternative treatments administered by providers or themselves (49). In addition, Chinese patients living in different regions (Shanghai, Hong Kong, Melbourne) have significant differences in their beliefs about seeking help for depression. For instance, people may seek help in the form of drugs, rather than talk therapy for depression (31). In other words, differences in ones’ social context (i.e., familial upbringing, education) also appears to affect people’s attitudes and preferences for seeking different types of help for depression. Hence, ethnic background and contextual factors associated with ones’ own and public attitudes toward a specific mental health problem and its treatment warrant consideration. Given this, we assert that non-psychiatric physicians must take into consideration ethnicity and social contexts when diagnosing depression, especially in the case of Chinese people, who may tend to express depression somatically (33) and seek help from non-psychiatric professionals (49).

Regardless of the mental health diagnosis, the study by Ben-David et al. (43) showed that family and friends have a critical influence in whether persons will seek professional help for their mental health problems. Further, in our study, the persons’ familial responsibilities could either impede or motivate them to seek medical help that might involve both a public and a financial cost. Similarly, Yu and colleagues (50) observed that family obligations prevented persons in China with depression from seeking medical support due to feelings of guilt about burdening their families and being incapable of carrying out their family duties. These results support the view that cultural influences relating to family context might hinder the disclosure of a mental illness, and put the ill member in a position of hesitating to seek familial support (51). However, we also found that family was a significant driving force when persons, especially younger individuals, hesitated to initiate a medical visit and/or continue follow-up treatment.

A study focused on the effect of cultural beliefs and practices on the recovery of individuals with psychiatric problems indicated that family involvement contributes to a better outcome for persons in developing countries than developed countries (52). The Chinese culture emphasizes that children are obligated to care for their parents in a kind and pleasant manner, in respect of the love and care that they received in their childhood (53). Therefore, we suggest that Chinese persons be reminded of their responsibility to their families, to motivate them to seek medical treatment, out of consideration for a future where they are able to fulfill their duty of being filial to their parents.

### 4.1. Implications

Most barriers preventing persons with mental health problems from seeking professional help were found to be similar to those in previous studies (21–24). For instance, previous studies identified the barriers as consisting of self-reliance, limited mental health literacy, lack of accessibility, distrust of physicians, stigma, opposition from others, unwillingness to disclose one’s condition, and a reluctance to burden one’s family (54, 55). In addition, similar to another study (56), many participants in our study stated that they lacked the motivation to seek treatment during their depressive episodes, since exhaustion commonly prevented them from seeking medical help. Indeed, people with depression are more likely than people without depression to report a reluctance to seek help (57). Hence, the impact of depressive symptoms themselves should be taken into consideration when health professionals adopt strategies to encourage persons to seek early treatment for their depression.

In terms of facilitators, initial support from family and friends can act as a significant motivator for persons to seek professional help and persist in treatment. In addition, persons might be encouraged to weigh the costs and benefits of medical treatment against stopping treatment and risking relapse, to encourage them to continue following through with medical treatment. Further, factors such as positive doctor-patient relations, education, and positive attitudes toward mental health services are important in persuading persons to access mental health services (55).

Early treatment and recovery for depression requires the first visit to a physician and/or psychiatrist about depression symptoms to be a positive one. Wheeler (58) also reported that being diagnosed with depression by practitioners was a meaningful start to the persons’ journey to initiate treatment. Hence, stepping up efforts to increase screening of depression in the general population may be helpful and necessary for its early detection. Moreover, educating the public about screening may expedite the process of persons receiving information that counteracts misassumptions about receiving a mental health diagnosis and care.

### 4.2. Limitations

As in all qualitative studies, one limitation is the generalizability of the study. However, readers are encouraged to discern whether the findings are transferable to similar Chinese groups with depression. Another limitation of the study is that we recruited participants from one outpatient department of a mental health center in an urban area of China; thus, those living in rural areas or those who have never visited a hospital are not represented in this study. The last limitation of this study is that most of the participants in our study were females and younger persons; therefore, male persons and those of an older age may have different experiences of seeking professional help. Therefore, we recommend that further studies involving these groups be conducted, as research on them is scarce.

## Data availability statement

The raw data supporting the conclusions of this article will be made available by the authors, without undue reservation.

## Ethics statement

The studies involving human participants were reviewed and approved by The Hong Kong Polytechnic University Ethics Committee Human Subjects Ethics Sub-Committee Departmental Research Committee. The patients/participants provided their written informed consent to participate in this study.

## Author contributions

JL, LH, and XL conducted the subject recruitment. YZ carried out the interviews and drafted the manuscript. LH and XL transcribed the recorded interviews verbatim. YZ, YM, and DL analyzed and interpreted the data. YM and DL critically reviewed and revised the manuscript. All authors conceived, designed the study, and approved the final draft of the manuscript.
